# Characterization of patient-derived HPV16 E6 and E7 variant alleles

**DOI:** 10.1128/jvi.00236-26

**Published:** 2026-03-30

**Authors:** Miranda Grace, Harshita Beeravolu, Ahmad Alhamshari, Josipa Skelin, Si-Young Kiessling, Andrew Bohm, Elizabeth A. White, Karl Munger

**Affiliations:** 1 Department of Developmental, Molecular, and Chemical Biology, Tufts University School of Medicine 12261, Boston, Massachusetts, USA; Dartmouth College Geisel School of Medicine, Hanover, New Hampshire, USA

**Keywords:** HPV16, E6 and E7 variant proteins, oncogenic mechanisms, protein–protein interactions, cervical carcinogenesis

## Abstract

Human papillomavirus type 16 (HPV16) is the most prevalent carcinogenic HPV type. Many HPV16 sequence variants have been described, some of which cause protein-coding mutations. However, most experiments have characterized the viral proteins encoded by the HPV16 prototype clone, and some researchers have proposed that variants identified in high-grade lesions and cancers may have higher oncogenic potential than the prototype. To determine whether oncoproteins encoded by such variants have greater oncogenic potential than the prototype version, we performed a comparative biochemical analysis of selected naturally occurring HPV16 E6 and E7 variant alleles identified in cervical lesions and/or control samples. We examined interactions of prototype and HPV16 E7 variant alleles with the retinoblastoma tumor suppressor pRB, the ubiquitin ligase UBR4 (p600), and the tumor suppressor PTPN14. We also compared HPV16 E6 prototype and variant protein interactions with the p53 tumor suppressor, the UBE3A (E6AP) ubiquitin ligase, and the PDZ-domain protein SCRIB. Several E6 or E7 variant proteins exhibited reduced binding to these cellular targets, suggestive of partial or complete loss of function. Our findings reveal that protein–protein interactions that enable the oncogenic activities of HPV16 E6 and E7 can be missing from naturally occurring HPV16 variants and that bona fide loss-of-function variants can be detected in high-grade cervical lesions or cancers. We conclude that clinical association alone is insufficient to predict the pathogenic potential of HPV16 E6 or E7 variant proteins.

**IMPORTANCE:**

Human papillomavirus type 16 (HPV16) is the leading cause of HPV-associated cancers, and most functional studies have been performed with the prototype viral clone. Numerous naturally occurring HPV16 variants have been detected in cervical lesions and cancers, raising the possibility that some variants may possess enhanced transforming activity. E6 and E7 are the major oncoproteins encoded by HPV16. By comparing key molecular interactions of prototype and naturally occurring E6 and E7 variant proteins, we found that several variant proteins display reduced engagement with tumor suppressors and ubiquitin ligases that are important for oncogenic activities. Notably, some variants found in high-grade lesions and cancers show partial or complete loss of engaging these critical host proteins. These findings indicate that the presence of an HPV16 variant in a cancer does not necessarily imply increased oncogenic potency. Functional characterization is therefore essential to interpret the biological and clinical significance of HPV16 genetic diversity.

## INTRODUCTION

Human papillomaviruses (HPVs) are a large group of epitheliotropic viruses with compact, ~8 kb double-stranded DNA genomes. Only a few of the more than 400 HPV types identified and sequenced (1, 2) are the high-risk types that are causally linked to cancers of the anogenital tract and oral mucosa (3). HPV is the most common sexually transmitted infection in the United States, causing more than 13 million new infections each year. Globally, cervical cancer is the fourth leading cause of cancer-related death in women, and approximately 5% of all human cancers are attributable to HPV infection (4, 5). While most HPV infections clear, approximately 10% of infected individuals develop persistent infections that, if undetected, place such individuals at a significantly increased risk for cervical cancer (3). HPV16 is the most prevalent high-risk HPV type and causes more than 50% of all cervical carcinoma and oropharyngeal cancers (3, 5, 6).

Despite the clinical importance of HPV16 infection, our understanding of the viral and host determinants that govern whether an HPV16 infection progresses to cancer remains limited. One reason for this knowledge gap is that most molecular studies have relied on the original HPV16 clone isolated from a cervical cancer by Harald zur Hausen’s group, often referred to as the prototype clone (7, 8). However, since that original isolation in 1983, many HPV16 variants have been identified. They comprise multiple lineages (A–D) and sublineages, each characterized by specific nucleotide polymorphisms and exhibiting distinct geographic distributions (9, 10). Infections with different HPV16 lineages and sublineages appear to confer different cancer risks (11–18). In the largest and most comprehensive investigation of HPV16 genetic variation to date, Mirabello et al. analyzed HPV16 genome sequences from 5,570 HPV16-positive cervical specimens collected from women with high-grade premalignant lesions or cervical cancer, as well as from women without detectable cervical abnormalities. They detected thousands of unique HPV16 genomes (19). Many variants likely arose because of a host cell antimicrobial defense mechanism, the upregulation of members of the APOBEC3 family of cytidine deaminases. APOBEC3-mediated deamination of cytosine residues to uracil generates characteristic C→G or C→T mutations, and many variants are consistent with this pattern (19). Some alterations caused amino acid changes in viral proteins including E6 and E7, which are the principal oncogenic drivers of HPV16-associated malignancies. Although some of the HPV16 E7 variant alleles have been biochemically and biologically characterized (20–22), the molecular consequences of many coding changes in HPV16 E6 or E7 remain largely unexplored. A central unanswered question is whether E6 or E7 variant alleles that are present in high-grade premalignant lesions or cervical cancer confer greater oncogenic activity than variant alleles identified primarily in HPV16-positive specimens from women without cervical disease.

To begin addressing this question, we assessed the association of HPV16 E6 and E7 prototype and variant alleles with several host cell target proteins and assessed E6, E7, and p53 steady-state levels. We chose eleven HPV16 E7 variant alleles and eleven HPV16 E6 variant alleles identified in cervical lesions or control samples (19). Several E6 and E7 variant proteins, regardless of disease association, exhibit reduced protein stability or impaired interactions with known cellular target proteins, suggesting that they may represent loss-of-function alleles. No correlation was found between the biological activity of HPV16 E6 and E7 variant proteins and their clinical associations.

## MATERIALS AND METHODS

### Plasmids and cells

Plasmids for transient transfection of HCT116 cells were generated by site-directed mutagenesis of CMV-C 16E7 (23) (Addgene #85035) or pNCMV 16E6no* (24) (Addgene #37454) using a QuikChange II site-directed mutagenesis kit (Agilent). Plasmids were prepared by Zymopure Plasmid Maxiprep (Zymo).

HCT116 (ATCC CCL-247) human colon carcinoma cells were cultured as recommended by ATCC. Human telomerase-immortalized normal oral keratinocytes (NOKs) (25) were cultured in keratinocyte serum-free medium (KSFM; Thermo Fisher Scientific/GIBCO).

### Transfection and retroviral transduction

HCT116 human colon cancer cells were transfected with E6 or E7 expression plasmids using polyethylenimine (PEI) (Polysciences) as described (26). At 48 h post-transfection, cells were lysed in EBC (50 mM Tris-HCl pH 8.0, 150 mM NaCl, 0.5% NP-40, and 0.5 mM EDTA) supplemented with protease inhibitors (Roche). Anti-HA Agarose (Sigma, A2095) was used for immunoprecipitations followed by SDS PAGE and western blot analysis on PVDF membranes. After incubation with the appropriate primary and secondary antibodies, protein bands were visualized by enhanced chemiluminescence and images acquired on a Syngene ChemiXX6 imager equipped with Genesys software version 1.5.5.0. All experiments were repeated multiple times with similar results (Table S1).

Retroviral vectors pLXSN16E6 (27) and mutants in the same backbone were used to generate HPV E6-expressing NOKs. G418-resistant cell pools were expanded, lysed, and processed for western blotting as described above.

### Antibodies

The following antibodies were used: PTPN14 (MAB4458, R&D), UBR4 (p600) (SC100615, Santa Cruz), pRB (BD554136, BD Biosciences), SCRIB (scribble) (4475, Cell Signaling), UBE3A (E6AP) (SC25509, Santa Cruz), p53 (ab32389, Abcam), Actin (MAB1501, EMD Millipore), and M2 Flag (F3165, Sigma). Secondary anti-mouse or anti-rabbit antibodies conjugated to horseradish peroxidase were from GE Healthcare and used according to their recommendations.

## RESULTS

### HPV16 E7 variant alleles

**Fig 1:**
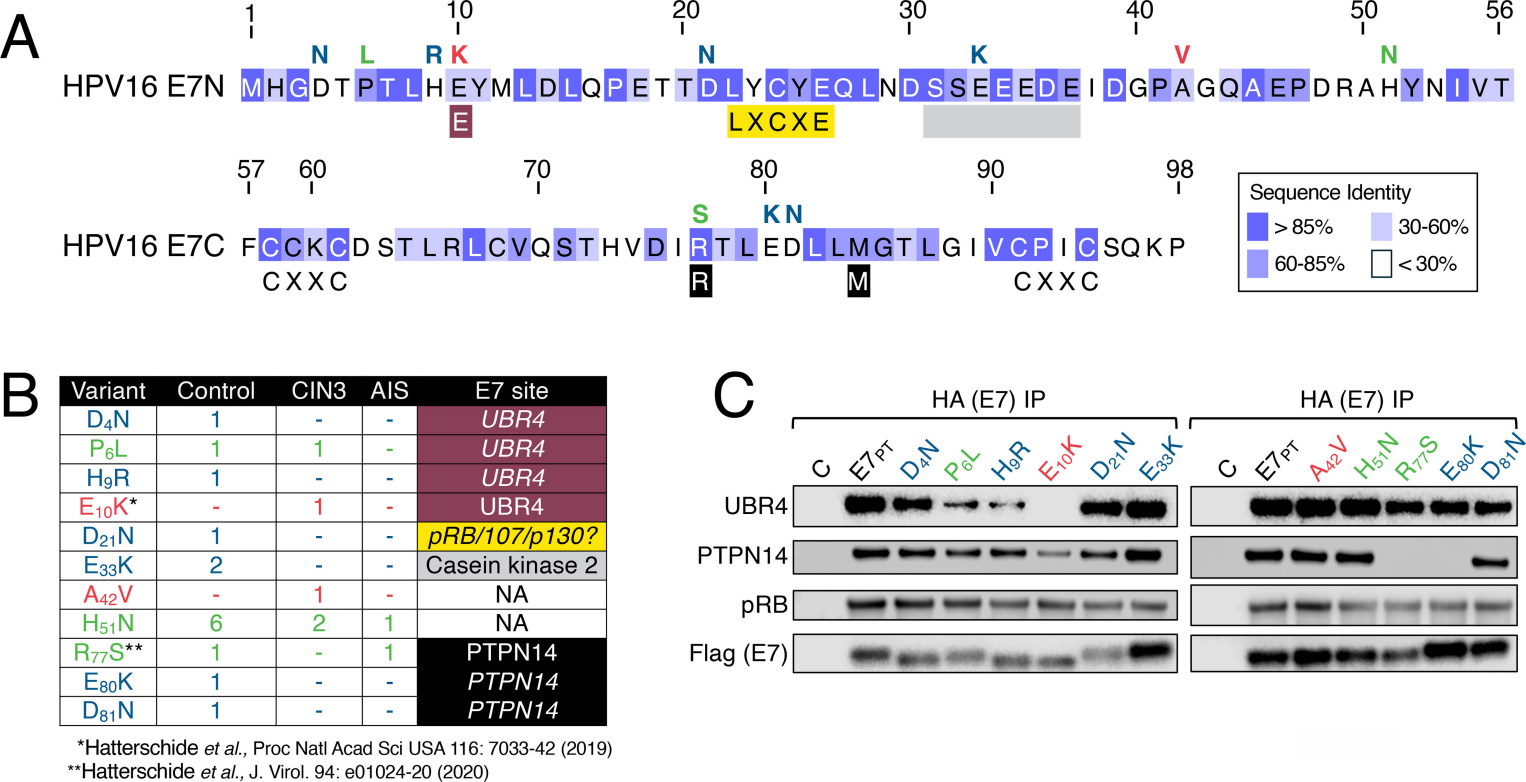
Biochemical activities of HPV16 E7 variants. (**A**) HPV16 E7 sequence. Blue shading indicates conservation of individual amino acids among high-risk HPV E7 proteins from HPV16, 18, 31, 33, 35, 39, 45, 51, 52, 56, 58, 59, 66, and 68 as indicated. The residues labeled under the full-length sequence are those known to be important for binding to UBR4 (E_10_) (20), pRB (LXCXE) (31), and PTPN14 (R_77_, M_84_) (21), and they are highlighted in purple, yellow, and black, respectively. The casein kinase 2 phosphorylation motif (48) is indicated by a gray bar, and the CXXC metal binding motifs are labeled. The residues labeled above the full-length sequence are the variants tested here. Red letters denote variants detected only in CIN3 lesions, blue letters are variants detected only in controls, and green letters denote variants that have been detected in controls and lesions (19). (**B**) Summary of the variants tested including the number of control, CIN3, or adenocarcinoma *in situ* (AIS) cases in which each variant was detected as well as the E7 site that may be altered. Predicted interaction sites are denoted in italics. NA, not applicable. Color coding as in panel **A**. (**C**) Binding of the prototype HPV16 E7 (E7_PT_) and the various E7 variants to UBR4, PTPN14, and pRB as assayed by transient transfection of HCT116 colon cancer cells. C-terminal Flag/HA-tagged HPV16 E7 prototype (E7_PT_) and variants were immunoprecipitated using HA antibody, and the precipitated E7 was detected by immunoblot using Flag antibody. Empty vector-transfected cells were used as controls (**C**). Color coding as in panel **A**. The blot shown is representative of multiple independent experiments. See Table S1 for details.

The high-risk HPV16 E7 protein is a major driver of carcinogenesis. Lacking intrinsic enzymatic activity, HPV16 E7 interacts with host proteins (6, 28–30) including the retinoblastoma tumor suppressor pRB (31), the ubiquitin ligase UBR4 (32, 33), and the non-receptor tyrosine phosphatase and tumor suppressor PTPN14 (34–36) to alter cellular signaling. UBR4 and pRB interact with sequences in the E7 N-terminus, whereas PTPN14 binds to conserved sequences in the E7 C-terminal domain (20, 21, 31–33, 37) (Fig. 1A). High-risk HPV16 E7 binding to pRB via the conserved LXCXE (L, leucine; C, cysteine; E, glutamic acid; and X, any amino acid) motif leads to pRB degradation (31, 38, 39), driving uncontrolled cell proliferation, a hallmark of cancer (40). In a separate complex, E7 binds to UBR4 and PTPN14. UBR4 binding contributes to anoikis resistance (32, 33, 41), likely because the E7-UBR4 complex targets PTPN14 for ubiquitination and degradation (34, 35). PTPN14 degradation results in activation of Yes-associated protein 1 (YAP1). YAP1 is a Hippo pathway transcriptional activator that promotes proliferation of epithelial progenitor cells, suppresses terminal differentiation, and coordinates cell shape, polarity, and tissue morphogenesis and regeneration (42–45). YAP1 activation driven by HPV16 E7-mediated PTPN14 degradation promotes retention of basal cell identity in infected keratinocytes and impairs their commitment to differentiation (21, 46, 47). High-risk HPV E7 mutants that lack the ability to bind pRB, UBR4, or PTPN14 are transformation defective (29).

Mirabello et al. identified 36 HPV16 E7 different coding variant alleles, only 5 of which were detected in cervical lesions or cancers (19). We selected eleven HPV16 E7 variant alleles for further analysis (Fig. 1B). This set included the five variant alleles detected in cervical lesions or cancers (P_6_L, E_10_K, A_42_V, H_51_N, and R_77_S). Among these, E_10_K and A_42_V were detected exclusively in a lesion. The others were detected both in lesions and in control samples from HPV16 positive, asymptomatic women (19). We also examined six variant alleles identified only in HPV16-positive control samples. These were chosen because the alterations from the prototype sequence were at amino acid residues close to known E7 interaction sites with UBR4 (D_4_N and H_9_R) (32, 33), pRB (D_21_N) (31), and PTPN14 (E_80_K and D_81_N) (21, 37), or within the casein kinase 2 motif (E_33_K) (48) (Fig. 1A and B).

The C-terminally HA–Flag–tagged HPV16 E7 variant alleles were generated by site-directed mutagenesis and cloned into the pCMV-Neo-Bam vector, followed by transient transfection into HCT116 colon carcinoma cells. These cells are highly transfectable, contain wild-type pRB and p53, and do not express exogenous viral sequences. Binding to UBR4, pRB, and PTPN14 was assessed by HA (E7) immunoprecipitation followed by immunoblotting with the corresponding antibodies. Flag antibody was used to detect immunoprecipitated E7. All 11 HPV16 E7 variant alleles produced detectable proteins. The HPV16 E7 D_4_N, D_21_N, E_33_K, A_42_V, H_51_N, and D_81_N variants bound pRB, UBR4, and PTPN14 at similar levels as prototype HPV16 E7. We previously reported that the HPV16 E_10_K variant protein does not bind UBR4 or degrade PTPN14 (20). Here we observed that the P_6_L and H_9_R variant proteins, which have alterations at adjacent sites, showed reduced binding to UBR4 (Fig. 1C). We also previously showed that the R_77_S variant allele is unable to bind or degrade PTPN14 (21). Similarly, the E_80_K variant allele was also PTPN14-binding deficient (Fig. 1C). By extrapolation from the structure of HPV18 E7 in complex with PTPN14 (37), HPV16 E7 R_77_ likely makes an electrostatic interaction with E_1095_ of PTPN14, and E_80_ is in the center of the E7-PTPN14 interface, where a charge reversal mutation would be expected to have a detrimental effect on binding.

In summary, several HPV16 E7 variant alleles, including P_6_L, E_10_K, and R_77_S, which were detected in cervical lesions, are defective for binding cellular target proteins and may represent loss-of-function alleles.

### HPV16 E6 variants

**Fig 2:**
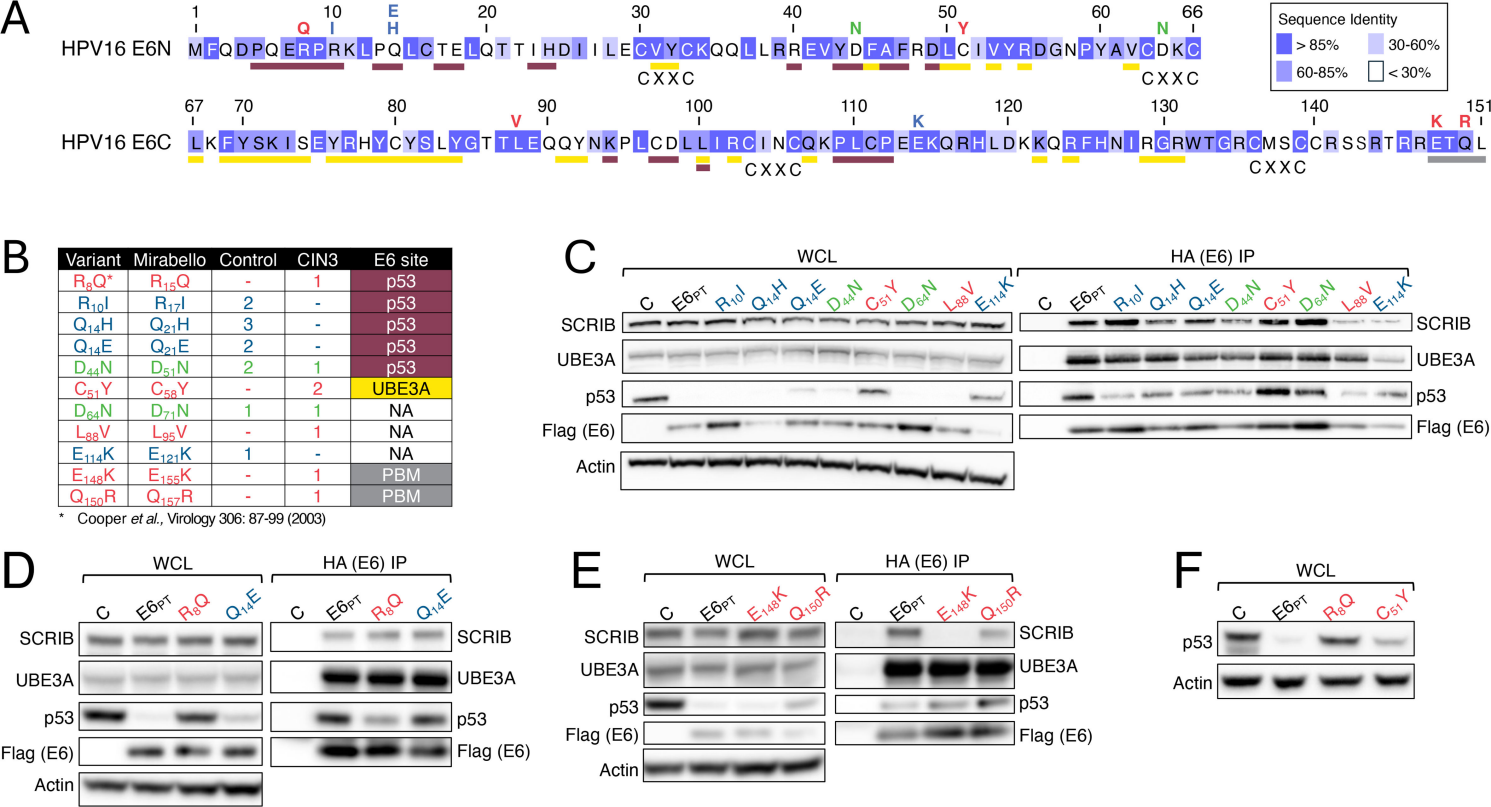
Biochemical activities of HPV16 E6 variants. (**A**) HPV16 E6 sequence. Blue shading indicates conservation of individual amino acids among high-risk HPV E6 proteins from HPV16, 18, 31, 33, 35, 39, 45, 51, 52, 56, 58, 59, 66, and 68 as indicated. The purple, yellow, and gray bars under the full-length sequence indicate the amino acids in the binding interfaces of E6 with UBE3A (64), p53 (64), and PDZ proteins (54, 55), respectively. The positions of the CXXC metal binding motifs are also indicated. The residues labeled above the full-length sequence are the variants tested here. Red letters denote variants detected only in CIN3 lesions, blue letters are variants detected only in controls, and green letters denote variants that have been detected in controls and in lesions (19). (**B**) Summary of the variants tested including the number of controls or CIN3 lesions where each variant was detected as well as the E6 site that may be altered. NA, not applicable. Color coding as in panel **A**. (**C–E**) Western blot analysis of the HPV16 E6 prototype (E6_PT_) and the variants tested. E6 steady-state levels and effects of E6 expression on SCRIB and p53 steady-state levels were assessed in whole cell extracts (WCL) of HCT116 colon cancer cells transiently transfected with N-terminal Flag/HA tagged prototype HPV16 E6 (E6_PT_) and the indicated E6 variants. An actin blot is shown as a loading control (left panels). Empty vector-transfected cells are shown as controls. Binding of prototype HPV16 E6 (E6_PT_) and E6 variants to the cellular PDZ protein SCRIB, the ubiquitin ligase UBE3A, and the p53 tumor suppressor was assayed by immunoprecipitation using HA antibody in the same cells. Precipitated E6 was detected by immunoblot using Flag antibody (right panels). Empty vector-transfected cells are shown as controls (**C**). Color coding as in panel **A**. (**F**) Effect of expression of untagged HPV16 E6 prototype (E6_PT_) and the E6 R_8_Q and C_51_Y variants on p53 steady-state levels after retroviral transduction of telomerase-immortalized NOKs (25). Empty vector-infected cells are shown as controls (**C**). Color coding as in panel (A). The results shown are representative of multiple independent experiments. See Table S1 for details.

The HPV16 E6 protein is also essential for HPV transformation (49, 50). Similar to E7, E6 lacks enzymatic activities and interacts with and functionally compromises or reprograms host cellular proteins (51). These include the tumor suppressor p53 (52), the ubiquitin ligase UBE3A (E6AP) (53), and cellular proteins containing PDZ (post synaptic density protein PSD95; *Drosophila* disc large tumor suppressor Dlg1; zonula occludens-1 protein ZO-1) domains (54, 55). PDZ proteins play diverse roles in inter- and intra-cellular signaling and trafficking (56) and high-risk HPV E6 proteins can interact with several cellular PDZ proteins (57), including the human homolog of the *Drosophila* tumor suppressor scribble, SCRIB (58). HPV16 E6 binds to a conserved LXXLL motif in UBE3A (59). The E6/UBE3A complex targets p53 for proteasomal degradation (60). Like other PDZ proteins, SCRIB has also been reported to be an E6/UBE3A degradation target (58, 61). Several structural studies of the HPV16 E6/UBE3A/p53 complex have been performed, and HPV16 E6 residues that are essential for p53 and UBE3A association have been identified (62–66). The structure of the C-terminal type I PDZ-binding motif (D/E–S/T–X–Φ, where Φ is a hydrophobic residue) of high-risk HPV E6 in complex with a PDZ domain peptide has also been determined (67). Unlike the PDZ binding motif, which is a short linear sequence at the E6 C-terminus, the amino acids that contact p53 or UBE3A are distributed throughout much of the E6 sequence, and these large interaction surfaces generate extremely high-affinity interactions (64) (Fig. 2A).

The HPV16 E6 open reading frame (ORF) contains two in-frame ATG codons that could initiate translation. The first ATG is at position 94 and the second ATG is at nucleotide 115. The E6 protein is translated from a bicistronic mRNA that is transcribed from a promoter at nucleotide 97 (P_97_) (68–70). Hence, the first ATG of the E6 ORF is not included on the E6/E7 mRNA and HPV16 E6 is translated from the ATG at nucleotide 115. However, Mirabello et al. annotated variant positions relative to the ATG at position 94 (19). In this report, we refer to E6 variant alleles with respect to the authentic initiation methionine at nucleotide 115 (Fig. 2B).

Given the large number of HPV16 E6 variant alleles discovered by Mirabello et al. (19), we focused on those altering highly conserved residues (E_114_K), residues involved in UBE3A binding (C_51_Y), p53-binding (R_8_Q, R_10_I, Q_14_E, Q_14_H, and D_44_N), or PDZ protein-binding (E_148_Q and Q_150_R), as well as other variant alleles detected in cervical lesions (D_64_N and L_88_V) (Fig. 2B). To assess interactions with these cellular proteins, N-terminally HA–Flag–tagged HPV16 E6 prototype and variant proteins were expressed in HCT116 cells. Whole-cell extracts were analyzed for E6, p53, and SCRIB steady-state levels. E6 binding to p53, UBE3A, and SCRIB was examined by HA (E6) immunoprecipitation followed by immunoblotting with the appropriate antibodies. Flag antibody was used to detect immunoprecipitated E6.

Some E6 variant alleles were expressed at lower levels than prototype HPV16 E6. These included the Q_14_H but not the Q_14_E variant protein (Fig. 2C and D) as well as the E_114_K (Fig. 2C) and Q_150_R (Fig. 2E) variant proteins. HPV16 E_148_K, and to a lesser extent Q_150_R, was defective for binding SCRIB. Even though SCRIB has been reported to be an E6/UBE3A degradation target (58), we did not detect differences in SCRIB steady-state levels in prototype versus SCRIB-binding-defective HPV16 E6-expressing cells (Fig. 2E). Consistent with earlier reports, the p53-binding-defective HPV16 E6 R_8_Q variant protein was defective for decreasing p53 steady-state levels (59). Similarly, the C_51_Y variant allele, in which a UBE3A contact residue is altered, was also defective for decreasing p53 steady-state levels even though we did not detect decreased UBE3A binding (Fig. 2C and D).

Having determined that the R_8_Q and C_51_Y variant alleles were markedly impaired in reducing p53 steady-state levels in the HCT116 transfection system, we tested the same variant alleles in a second experimental format. Recombinant retroviruses expressing untagged HPV16 E6 prototype and the R_8_Q and C_51_Y variant proteins were used to infect telomerase-immortalized normal human oral keratinocytes (NOKs), which is a biologically relevant cell type (25). After selection, p53 steady-state levels were assessed by western blotting. Prototype HPV16 E6 and control vector expressing cells were used as positive and negative controls, respectively. Consistent with the results obtained in transiently transfected HCT116 cells (Fig. 2C and D), HPV16 E R_8_Q, and to a lesser extent C_51_Y, showed defects in reducing p53 steady-state levels (Fig. 2F).

In conclusion, as with E7, several E6 variants, including R_8_Q, C_51_Y, E_148_K, and Q_150_R that were detected in high-grade cervical lesions are potential loss-of-function alleles.

## DISCUSSION

Several prior reports on variants have suggested that cancer-associated HPV16 variants may be more oncogenic than the prototype clone (11–18). We found that several HPV16 E6 and E7 variant alleles that were detected in cervical lesions were defective for binding cellular target proteins and thus may represent loss-of-function alleles. Other E6 and E7 variant alleles isolated from high-grade lesions or controls could bind to all the host cell targets we tested, and a third set of E6 variant alleles detected in controls encoded unstable proteins and therefore may also represent loss-of-function alleles. In summary, we did not observe a correlation between clinical association and the biochemical activities that we tested.

The lack of such a correlation is in contrast to the conclusions of another study by Lou et al., where soft agar growth and wound-healing assays in NIH3T3 fibroblasts were used as a predictor of HPV16 E7 oncogenic potential (22). Unlike ours, those results suggested that many of the HPV16 E7 variants detected in controls had reduced transforming activity, whereas lesion-associated variants transformed at similar levels as the prototype HPV16 E7 protein. Both studies assessed variants in two regions of E7, the N-terminal UBR4 binding region and the C-terminal PTPN14 binding site. Related to the PTPN14 binding site, Lou et al. showed that the HPV16 E7 R_77_S variant, which was detected in an adenocarcinoma *in situ*, transformed NIH3T3 cells similar to prototype HPV16 E7 (22). In contrast, Hatterschide et al. demonstrated that the PTPN14-binding defective HPV18 R_84_S mutant, which corresponds to the HPV16 E7 R_77_S variant allele, failed to extend the lifespan of primary human foreskin keratinocytes (21). These contrasting results highlight the importance of studying HPV E7 oncogenic potential in biologically relevant human cells that express all of the host cell proteins targeted by E7.

Related to the UBR4 binding site, Lou et al. reported that the H_9_R variant, which was detected in a control, was transformation-defective. In contrast, P_6_L, detected in a CIN3 lesion and a control, transformed NIH3T3 cells equivalent to the HPV16 E7 prototype (22). Here, we tested the same variant proteins and found that P_6_L and H_9_R are both defective in UBR4 binding. We also previously found that UBR4-binding-deficient HPV16 E7 E_10_K variant allele, which was detected in a CIN3 lesion, was defective for human foreskin keratinocyte lifespan extension (20). HPV16 E7 E_10_K was not tested by Lou et al. Neither our experiments here nor the biological experiment in primary keratinocytes (20, 21) demonstrated a correlation between clinical association and HPV16 E7 oncogenicity.

While we did not perform any biological assays with the HPV16 E6 variant alleles, our study suggests that several have characteristics of loss-of-function alleles. Some of the coding changes caused HPV16 E6 to be expressed at lower levels. Of the 11 variant proteins we evaluated, 3 were consistently present at lower levels. These are Q_14_H, E_114_K, and Q_150_R, and they were found in three controls, one control, and a CIN3 lesion, respectively. Other variant proteins were defective for p53 degradation. These are HPV16 R_8_Q, which alters a residue involved in p53 binding and was previously identified as p53 binding and degradation defective in a yeast-based random mutagenesis screen (59), and HPV16 C_51_Y, which alters a residue involved in UBE3A binding. C_51_Y consistently had a more modest effect on p53 levels than R_8_Q, but both these variant alleles were detected in CIN3 lesions. Still others were defective for binding to the PDZ protein SCRIB. These include HPV16 E_148_K and HPV16 Q_150_R which alter sequences within the C-terminal PDZ binding site. These were also detected in CIN3 lesions. We conclude that similar to the E7 variant alleles that we analyzed, there was no correlation between these biochemical activities of E6 and clinical association. Several E6 variant alleles detected in cervical lesions likely represent loss-of-function alleles.

Our study has some limitations. First, the experimental format that we used may lack the sensitivity to detect E6 or E7 variant alleles that might bind and/or degrade cellular targets more efficiently than the prototype proteins. Our study, therefore, does not rule out that hyperactive HPV E6 or E7 variant alleles exist. Second, it is unclear from the original study whether some specimens contained HPV16 variants that harbored combinations of coding changes in one or several viral open reading frames (19). Such combinations could give rise to epistatic interactions that are not captured by the single-mutation analyses that we performed here. Third, in many cases, the variant genome was not the only viral species detected, and it therefore cannot be ruled out that residual wild-type HPV16 genomes contribute to the biological phenotype of the infected tissue. However, in most samples, the minor allele frequency exceeded 60% of sequencing reads (19). Such a high prevalence of a variant viral genome within a tissue is consistent with evolutionary fitness and suggests positive selection, although additional functional validation would be required to test this. Fourth, although binding to the cellular targets of E6 and E7 that we assessed here is well-established to correlate with transformation (29, 51, 71), some variant proteins may be defective for binding other host proteins that also contribute to oncogenic activity.

On the other hand, the fact that host protein-binding-defective HPV16 E6 and E7 variant alleles are detected in high-grade lesions or cancers does not imply that these interactions are unrelated to oncogenic potential. Rather, such variant alleles could arise in individuals (or lesions) with a specific genetic background. For example, an analysis of whole-genome and whole-exome sequencing data from Icelandic and UK Biobank cohorts revealed that high-penetrance germline *PTPN14* variants are associated with a strong genetic predisposition for cervical cancer development (72). Related to our findings here, it is enticing to speculate that individuals who developed cervical lesions harboring the PTPN14 degradation–defective HPV16 E_10_K and/or R_77_S variant alleles carried such PTPN14 mutant alleles. Thus, future studies will benefit from considering the genetic makeup of the host when evaluating the carcinogenic potential of individual HPV variants.

## Data Availability

Plasmids generated in this study are made available through Addgene (https://www.addgene.org). All data supporting the findings of this study are available within the article and its supplemental material.

## SUPPLEMENTAL MATERIAL

Table S1 jvi.00236-26-s0001.docx Number of times experiments were repeated.

ASM does not own the copyrights to Supplemental Material that may be linked to, or accessed through, an article. The authors have granted ASM a non-exclusive, world-wide license to publish the Supplemental Material files. Please contact the corresponding author directly for reuse.
