# Supplementary material for: Characterization of patient-derived HPV16 E6 and E7 variant alleles
Source: J Virol. 2026 Mar 30;100(4):e00236-26. doi: 10.1128/jvi.00236-26 (PMC13098253; doi:10.1128/jvi.00236-26)
Supplement: Table S1 — Number of times experiments were repeated. [file jvi.00236-26-s0001.docx]

Table S1:

Number of independent experiments performed to reproduce the results shown in Figures 1 and 2

| HPV16 E7 variant protein | # of experiments |
| --- | --- |
| D_4_N | 3 |
| P_6_L | 3 |
| H_9_R | 3 |
| E_10_K | 3* |
| D_21_N | 3 |
| E_33_K | 3 |
| A42V | 3 |
| H51N | 3 |
| R77S | 3** |
| E80K | 8 |
| D81N | 3 |

Independently tested:

* Hatterschide *et al.,* Proc Natl Acad Sci USA 116: 7033-42 (2019)

** Hatterschide *et al.*, J. Virol. 94: e01024-20 (2020)

| HPV16 E6 variant protein | # of experiments |
| --- | --- |
| R_8_Q | 3* |
| R_10_I | 4 |
| Q_14_H | 4 |
| Q_14_E | 4 |
| D_44_N | 4 |
| C_51_Y | 6 |
| D_64_N | 3 |
| L_88_V | 3 |
| E_114_K | 3 |
| E_148_K | 8 |
| Q_150_R | 5 |

Independently discovered:

* Cooper *et al.,* Virology 306: 87-99 (2003)
